# Thyroglossal Duct Cysts in the Adult Population: A Retrospective Analysis of Clinical Characteristics and Outcomes

**DOI:** 10.7759/cureus.107988

**Published:** 2026-04-29

**Authors:** Maria C Torres Gonzalez, Carlos Alfredo Bautista Lopez, Carla M Cruz Rocha, Marco A Nava Romero, David J Alvarez Chavez, Daniela I Sanchez Lozano

**Affiliations:** 1 General Surgery, Centro Universitario de Ciencias de la Salud, Universidad de Guadalajara, Guadalajara, MEX; 2 General Surgery, Hospital Civil de Guadalajara "Dr. Juan I. Menchaca", Guadalajara, MEX

**Keywords:** head and neck tumors and diseases, otolaryngology-head & neck surgeons, sistrunk procedure, thyroglossal cyst, thyroglossal duct cyst malignancies

## Abstract

Background

Thyroglossal duct cysts (TGDCs) represent the most common congenital midline neck mass; however, their presentation in adults is uncommon and often underreported. Adult cases may differ clinically from pediatric cases in symptomatology, diagnostic pathways, and risk of associated malignancy. This study contributes to the limited literature describing TGDCs in adults and highlights the importance of documenting their presentation and outcomes in this age group.

Objective

To describe the demographic, clinical, surgical, and histopathological characteristics of adult patients with TGDCs treated in a tertiary care center and to evaluate postoperative outcomes following the Sistrunk procedure.

Methods

A retrospective observational study was conducted including all adult patients aged ≥18 years who underwent surgical management for TGDC between May 2017 and November 2025 at the General Surgery Service of Hospital Civil de Guadalajara “Dr. Juan I. Menchaca.” Variables analyzed included demographics, clinical features, preoperative imaging, surgical technique, intraoperative findings, histopathological results, and postoperative evolution. All patients were treated with the standard Sistrunk procedure. Descriptive statistics were obtained using IBM SPSS v27. The mean follow-up duration was 2 months.

Results

Nine patients met the inclusion criteria (n=9). The cohort consisted of females (n=5, 55.6%) and males (n=4, 44.4%), with most individuals aged 40-60 years (n=5, 55.6%). Comorbidities were present in 3 patients (33.3%), including papillary thyroid carcinoma (n=1, 11.1%), hypothyroidism (n=1, 11.1%), and hypertension (n=1, 11.1%). All patients presented with a midline neck mass (n=9, 100%), and 3 patients (33.3%) reported intermittent enlargement and regression. The Sistrunk procedure was performed in all cases (n=9, 100%) without intraoperative or immediate postoperative complications (n=0, 0%). The mean cyst diameter was 3.4 cm (range: 1.2-5.6 cm). Histopathology identified ectopic thyroid tissue in 2 patients (22.2%), while no cases demonstrated malignancy (n=0, 0%). No patient developed postoperative hypothyroidism (n=0, 0%), and no recurrence was observed during the study period.

Conclusion

TGDCs in adults exhibit a broad age distribution, underscoring the importance of maintaining diagnostic suspicion even beyond childhood. The Sistrunk procedure remains a safe and effective treatment in adults, with excellent outcomes. Documentation of TGDCs in adult populations is essential to better characterize their behavior, refine diagnostic approaches, and assess the true prevalence of associated comorbidities or malignancy in this underrepresented group.

## Introduction

Congenital masses of the anterior neck comprise a heterogeneous group of lesions predominantly identified during childhood. Among these, thyroglossal duct anomalies represent the most common entity, with an estimated prevalence of approximately 7% in the general population based on autopsy studies and a female-to-male ratio of 2:1 [1,2]. These lesions typically present as midline cervical swelling, most frequently located in the suprahyoid region, representing 60-80% of cases, although they may arise at any point along the embryological tract of the thyroglossal duct, including the base of the tongue (2.1%) and the suprasternal region (12.9%) [2,3].

Despite their congenital origin, presentation in adulthood is relatively uncommon and remains underreported. Most of the available literature focuses on pediatric populations, and adult-specific data regarding clinical presentation, management, and outcomes remain limited, highlighting the need for further characterization in this group [4-6].

The coexistence of synchronous thyroid malignancy is rare, as the vast majority of these lesions are benign. Approximately 1-1.5% of cases are associated with carcinoma, most commonly papillary carcinoma (~80%), followed by follicular or mixed variants (~9%) [1,4].

Clinically, most patients remain asymptomatic, and the presence of a painless midline neck mass is the most frequent reason for consultation. Nevertheless, between 22% and 43% of patients may present with dysphagia or recurrent upper respiratory tract infections [2,3].

From an embryological perspective, the thyroglossal duct represents the remnant of the thyroid gland’s descent from the foramen cecum to its final pretracheal position. This tract may remain closely associated with the hyoid bone, explaining its anatomical variability, and typically involutes between the eighth and tenth weeks of gestation [4,6].

Histologically, thyroglossal duct cysts (TGDCs) are lined by squamous, columnar, or pseudostratified ciliated epithelium and may contain ectopic thyroid tissue. Cyst formation is attributed to recurrent inflammation and accumulation of secretions [5-7].

Cervical USG is the diagnostic modality of choice, typically demonstrating a well-defined midline cystic lesion, while computed tomography is reserved for selected or complex cases [8].

Surgical management remains the treatment of choice. The Sistrunk procedure, comprising en bloc resection of the cyst, the central portion of the hyoid bone, and the tract, has reduced recurrence rates to approximately 0-12.2% (mean ~4%), compared with up to 22% following simple excision [9,10].

## Materials and methods

A retrospective, descriptive, observational study was conducted, including all adult patients who underwent surgical treatment for TGDCs between May 2017 and November 2025 at the General Surgery Department of the Dr. Juan I. Menchaca Civil Hospital in Guadalajara. Patients aged 18 years or older with a clinical and imaging diagnosis compatible with a TGDC who underwent the Sistrunk procedure and for whom a confirmatory histopathological report was available were included. Patients with histopathological diagnoses other than TGDCs, incomplete surgeries, surgeries performed outside the department, and records with insufficient information for analysis were excluded.

Demographic, clinical, surgical, histopathological, and follow-up variables were collected. Demographic variables included patient age and sex. Clinical variables included the reason for consultation, including increased volume, compressive symptoms, previous infection, or changes in size; duration of symptoms; relevant medical history, such as thyroid pathology, previous infections, or comorbidities; imaging studies used, primarily ultrasound and, in selected cases, computed tomography; and the presence or absence of ectopic thyroid tissue. Surgical variables included the technique used, corresponding to the standard Sistrunk procedure, intraoperative findings, cyst size in centimeters, and both intraoperative and immediate postoperative complications, defined as any event occurring within 30 days of surgery. Histopathological variables included diagnostic confirmation of a TGDC, presence of ectopic thyroid tissue identified in the cyst wall, evidence of infection or chronic inflammation, and identification of malignancy. During follow-up, recurrence, defined as the clinical reappearance of a midline neck mass confirmed by ultrasound, development of hypothyroidism, the need for reintervention, and synchronous thyroid findings were evaluated.

All patients were initially evaluated by cervical ultrasound performed by an expert radiologist, while computed tomography and other studies were reserved for cases with atypical characteristics, a suspected extensive tract, or a history of recurrence. The surgical approach consisted of performing the Sistrunk procedure by attending surgeons of the service, or under their direct supervision, all of whom were board-certified general surgeons, through a transverse cervical incision, dissection of the cyst and its tract to its insertion in the hyoid bone, resection of the middle segment of the hyoid bone, and continuation of the dissection toward the remaining tract in the direction of the foramen cecum, culminating in careful hemostasis and layered closure.

The data obtained were recorded in an electronic database using Google Sheets and analyzed with IBM SPSS Statistics version 27.0. Frequencies and percentages were used for qualitative variables, and means and ranges were used for quantitative variables. Follow-up duration ranged from 1 to 6 months, with a median of 2 months. The study was conducted in accordance with the Declaration of Helsinki and current national regulations for research involving human subjects, ensuring complete anonymization of the information. It was classified as minimal-risk research because it was a retrospective analysis without additional intervention in the patients.

## Results

Nine patients with a histopathologically confirmed diagnosis of TGDC were included (n=9, 100%).

The sex distribution showed a female predominance (n=5, 55.6%) compared with male patients (n=4, 44.4%). The mean age was 41 ± 15 years, with the highest concentration in the 40-60-year age group (n=5, 55.6%), followed by the 20-40-year age group (n=2, 22.2%), the >60-year age group (n=1, 11.1%), and the 16-20-year age group (n=1, 11.1%).

Analysis of medical history revealed that comorbidities were present in 3 patients (33.3%), including papillary thyroid carcinoma (n=1, 11.1%), hypothyroidism (n=1, 11.1%), and systemic hypertension (n=1, 11.1%), while the remaining patients (n=6, 66.7%) reported no relevant medical conditions. The reason for consultation was uniform, as all patients presented with a midline neck mass (n=9, 100%). Additionally, a subgroup of patients (n=3, 33.3%) reported a fluctuating clinical course characterized by intermittent enlargement and regression in mass size, suggesting possible inflammatory episodes or variable cystic content retention. Table 1 summarizes the demographic and clinical characteristics of the study population.

**Table 1:** Demographic characteristics of patients with thyroglossal duct cysts (n = 9).

Variable	Value
Total number of patients	9
Female	5 (55.6%)
Male	4 (44.4%)
Age range, 16-20 years	1 (11.1%)
Age range, 20-40 years	2 (22.2%)
Age range, 40-60 years	5 (55.6%)
Age range, >60 years	1 (11.1%)
Mean age	41 ± 15 years

The Sistrunk procedure was performed in all cases (n=9, 100%), with no intraoperative complications (n=0, 0%) or immediate postoperative complications (n=0, 0%), reflecting appropriate patient selection and standardization of the surgical technique. The mean cyst diameter was 3.4 cm (range: 1.2-5.6 cm).

Histopathological analysis revealed the presence of ectopic thyroid tissue in 2 patients (22.2%), while no cases of malignancy were identified (n=0, 0%). Table 2 summarizes the surgical and histopathological findings.

**Table 2:** Histopathological results and follow-up. TGDC: Thyroglossal duct cyst.

Variable	Outcome
Confirmed diagnosis of TGDC	100%
Ectopic thyroid tissue	2 patients (22.2%)
Malignancy	0%
Development of hypothyroidism	0%
Recurrence	0%
Reintervention	0%

It is noteworthy that none of the patients developed postoperative hypothyroidism (n=0, 0%), including those with ectopic thyroid tissue or prior thyroid disease, suggesting that surgical resection did not negatively impact overall thyroid function during follow-up. Follow-up duration ranged from 1 to 6 months, with a median of 2 months.

## Discussion

The presentation of TGDC in adults poses a clinical and diagnostic challenge, as it differs in several aspects from that in pediatric populations. Although TGDC is the most common congenital cervical anomaly, its diagnosis at older ages has become increasingly frequent due to the widespread use of imaging studies and greater clinical awareness [2,6,11]. Various series have shown that although most cases are detected during childhood, a significant proportion are identified in adults, including patients in the seventh decade of life [8,12,13]. This age variability is reflected in contemporary cohorts documenting bimodal patterns of presentation, suggesting that persistent epithelial remnants may remain asymptomatic for decades before becoming clinically evident [3,14,15].

**Figure 1:**
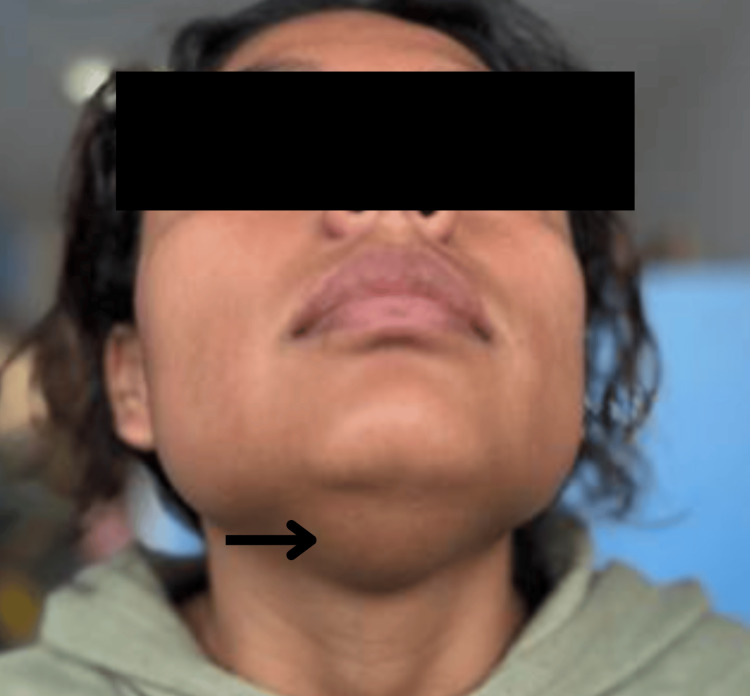
Thyroglossal duct cyst showing focal soft-tissue swelling in the central region of the neck, frontal view.

Consistent with the literature, the clinical presentation in adults typically includes a midline cervical mass that moves with swallowing and tongue protrusion. However, adults tend to be more symptomatic than pediatric patients, more frequently presenting with inflammation, pain, fluctuating size, and compressive symptoms. These findings were also observed in our population, where focal soft-tissue swelling was evident in the anterior cervical region (Figure 1) [8,9,16]. Tan SW et al. described large TGDCs extending from the submandibular region to the suprasternal area, causing dysphagia or dyspnea [10]. This clinical variability underscores the importance of a broad differential diagnosis, including dermoid cysts and cystic metastases of papillary thyroid carcinoma, particularly in the presence of lateralization, rapid growth, or suspicious imaging features [17,18].

**Figure 2:**
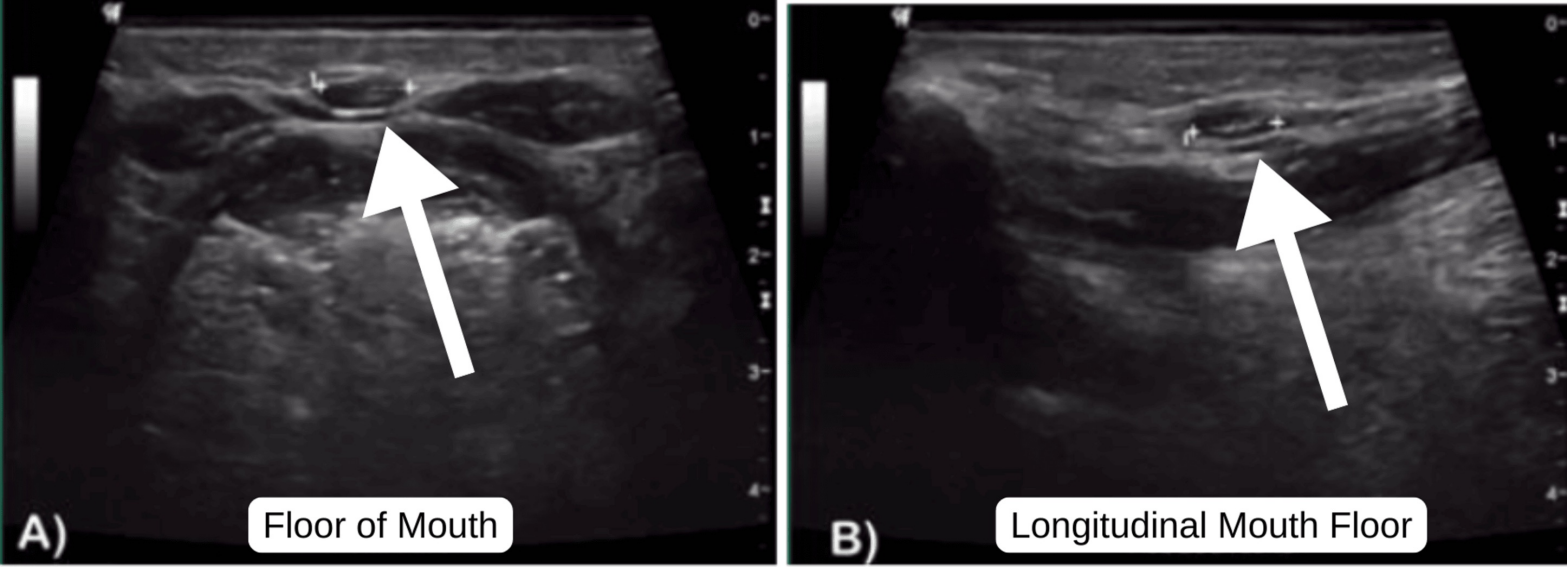
Cervical ultrasound showing an anechoic lesion with regular borders and a thin wall at the infrahyoid level. Transverse (A) and longitudinal (B) views are shown.

Imaging evaluation constitutes a fundamental pillar in diagnosis. Ultrasound remains the initial study of choice due to its availability and high sensitivity for identifying cystic lesions, as well as for assessing ectopic thyroid tissue (Figure 2) [2].

**Figure 3:**
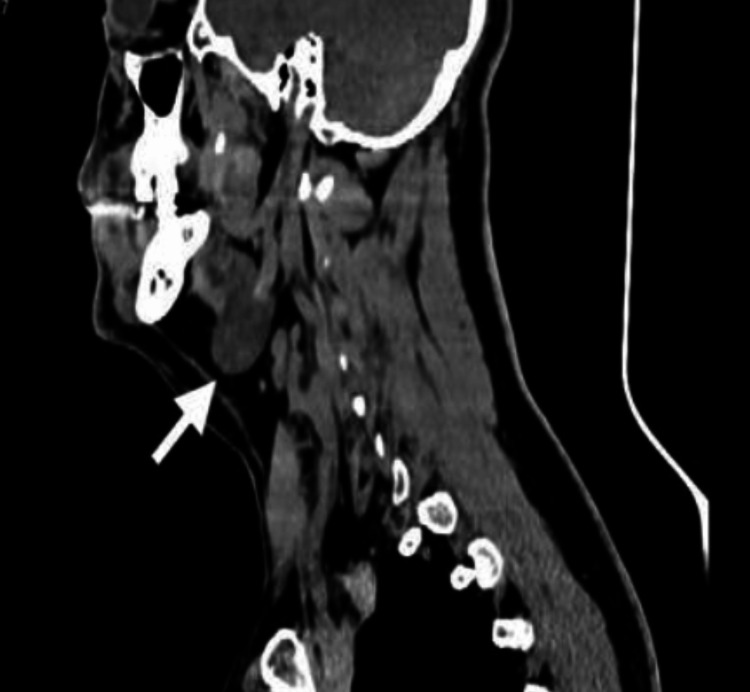
Sagittal CT scan showing a well-defined hypodense lesion with regular borders in the submandibular region.

In adults, the increased suspicion of malignancy leads to more frequent use of computed tomography and magnetic resonance imaging. Bhama AR et al. reported significant differences in preoperative diagnostic approaches between adult and pediatric populations [4,19]. Computed tomography may demonstrate a well-defined hypodense lesion, as illustrated in Figure 3. Additionally, when imaging reveals solid or irregular components, fine-needle aspiration (FNA) may be considered; however, its sensitivity is limited, and definitive diagnosis relies on histopathological examination [7,11,18].

A distinctive aspect in adults is the importance of diagnosing and managing malignancy. Although carcinoma arising within TGDC is rare, its incidence is relatively higher in adults, with most cases corresponding to papillary carcinoma [1,19,20]. Alatsakis M et al. described invasive cases with extracystic extension, emphasizing the need for comprehensive evaluation when suspicious features are present [1]. Surgical decision-making depends on tract involvement, coexistence of thyroid carcinoma, and other prognostic factors; in selected cases, total thyroidectomy in addition to the Sistrunk procedure may be required [1,21].

**Figure 4:**
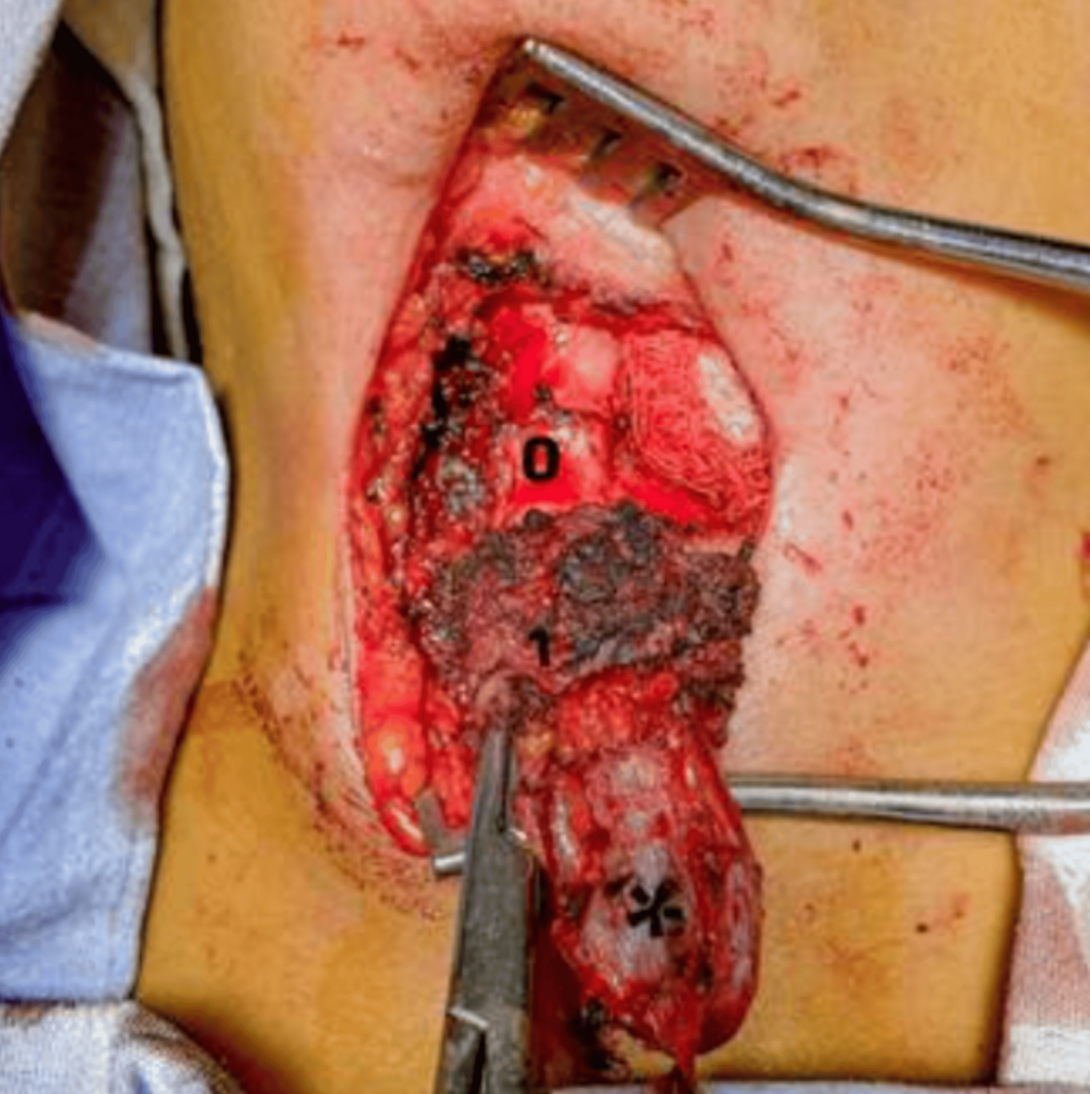
Intraoperative image of the Sistrunk procedure showing the thyroglossal duct (0), resected central portion of the hyoid bone (1), and thyroglossal duct cyst (*).

The standard surgical approach remains the Sistrunk procedure, recognized as the most effective method for reducing recurrence, with rates ranging from 3% to 11% in large series [15,18,22,23]. Comparative studies, such as those by Rattan KN et al. and Zaman SU et al., confirm that the Sistrunk technique provides superior outcomes compared with simple excision, particularly in reducing recurrence and complications [14,16]. Factors such as prior infection or inadequate initial surgery have been associated with increased recurrence risk, highlighting the importance of appropriate preoperative management [8,11,24]. In our series, intraoperative anatomy and key surgical landmarks were clearly identified, as shown in Figure 4, reinforcing the importance of complete excision of the duct and central portion of the hyoid bone.

In adults, modifications of surgical techniques have also been proposed to improve cosmetic outcomes, which are particularly relevant in this population. Rattan KN et al. described alternative incision approaches that enhance aesthetic results without compromising complete tract excision [14]. In cases of multiple recurrences, O'Neil LM et al. proposed extended anterior neck dissection, demonstrating favorable outcomes in complex cases [23].

Long-term follow-up remains essential, particularly in adults, given the higher likelihood of incidental findings and potential malignant transformation. Kim SC et al. demonstrated that incidentally detected TGDCs in adults may remain stable over time, suggesting that conservative management may be appropriate in selected high-risk surgical patients [12]. This approach is supported by recent studies evaluating non-surgical management in elderly populations [11,25].

Taken together, the available evidence indicates that TGDC in adults presents distinct clinical, diagnostic, and therapeutic characteristics compared with pediatric populations. The integration of advanced imaging, careful assessment of malignancy risk, and appropriate surgical planning is fundamental for optimal management. The Sistrunk procedure remains the gold standard, offering low recurrence rates and excellent functional and cosmetic outcomes when performed under appropriate conditions.

This study has several limitations that should be considered when interpreting the findings. The relatively small sample size (n=9) limits the ability to perform inferential statistical analysis. Additionally, the retrospective, single-center design may introduce selection bias and limit external validity. The absence of a comparison group restricts direct evaluation against alternative management strategies or different age populations. Nevertheless, these findings provide meaningful clinical insight into an underrepresented adult population and contribute to the growing body of literature on the presentation and surgical outcomes of TGDCs in adults.

## Conclusions

This study demonstrates that TGDC should not be considered exclusively a pediatric condition, as it can also present in adults with notable frequency. In our series of nine adult patients with histopathologically confirmed TGDC, we observed a broad age distribution and a predominance of female patients, consistent with the existing literature. The absence of postoperative complications, lack of recurrence during follow-up, and satisfactory cyst-related outcomes support the safety and effectiveness of the Sistrunk procedure as the treatment of choice. Additionally, the presence of ectopic thyroid tissue in some cases highlights anatomical variability in adults; importantly, its resection did not lead to postoperative thyroid dysfunction, further supporting a conservative surgical approach.

Reporting adult TGDC cases remains clinically important, as it raises awareness of this condition beyond childhood and helps prevent diagnostic delays. It also enables systematic evaluation of surgical outcomes and contributes to a clearer understanding of the risk of malignant transformation, which, although rare, has been documented. Overall, these findings support maintaining a high index of suspicion in adults with midline neck masses and reinforce the Sistrunk procedure as a safe and effective standard of care, while emphasizing the need for continued surveillance and further case reporting.

## References

[1] Alatsakis M, Drogouti M, Tsompanidou C (2018). Invasive thyroglossal duct cyst papillary carcinoma: a case report and review of the literature. Am J Case Rep.

[2] Al-Thani H, El-Menyar A, Sulaiti MA (2016). Presentation, management, and outcome of thyroglossal duct cysts in adult and pediatric populations: a 14-year single-center experience. Oman Med J.

[3] Barber J, Martinez DS, Palma DF, Stark AP, Livhits MJ (2018). Intrathyroidal thyroglossal duct cyst: a rare cause of thyroiditis in an adult. AACE Clin Case Rep.

[4] Bhama AR, Smith RJ, Robinson RA, Weigel RJ, Sugg SL, Howe JR, Lal G (2014). Preoperative evaluation of thyroglossal duct cysts: children versus adults--is there a difference?. Am J Surg.

[5] Hassan E, See GB, Aziz DA (2014). Thyroglossal duct cysts: a ten years retrospective review. Egypt J Ear Nose Throat Allied Sci.

[6] Ndegbu CU, Olasehinde O, Adeyemo A, Alatise OI, Amusa YB (2021). Management of thyroglossal cyst in adults: a single-institution experience. Niger J Surg.

[7] Booth R, Tilak AM, Mukherjee S, Daniero J (2019). Thyroglossal duct cyst masquerading as a laryngocele. BMJ Case Rep.

[8] Soni S, Poorey VK, Chouksey S (2014). Thyroglossal duct cyst, variation in presentation, our experience. Indian J Otolaryngol Head Neck Surg.

[9] Eshraghi R, Gupta D (2024). A thyroglossal duct cyst presenting in an adult. Int J Case Rep Clin Images.

[10] Tan SW, Misron K, Tengku Kamalden TM (2023). Large thyroglossal duct cyst presenting in adulthood: a case report and literature review. Cureus.

[11] Pomponio MK, Conti KR, Ohlstein JF, Khan I, Koch T (2024). Thyroglossal duct cysts (TGDC) in the elderly population: the role of conservative management. Cureus.

[12] Kim SC, Sun HY, Kim HS, Ryoo I (2018). Long-term ultrasound follow-up of incidentally detected thyroglossal duct cysts in adults. AJNR Am J Neuroradiol.

[13] Mortaja S, Sebeih H, Alobida NW, Al-Qahtani K (2020). Large thyroglossal duct cyst: a case report. Am J Case Rep.

[14] Rattan KN, Kalra VK, Yadav SP, Vashist A, Vashisth S (2020). Thyroglossal duct remnants: a comparison in the presentation and management between children and adults. Indian J Otolaryngol Head Neck Surg.

[15] Oomen KP, Modi VK, Maddalozzo J (2015). Thyroglossal duct cyst and ectopic thyroid: surgical management. Otolaryngol Clin North Am.

[16] Zaman SU, Ikram M, Awan MS, Hassan NH (2017). A decade of experience of management of thyroglossal duct cyst in a tertiary care hospital: differentiation between children and adults. Indian J Otolaryngol Head Neck Surg.

[17] Patel S, Bhatt AA (2019). Thyroglossal duct pathology and mimics. Insights Imaging.

[18] de Tristan J, Zenk J, Künzel J, Psychogios G, Iro H (2015). Thyroglossal duct cysts: 20 years' experience (1992-2011). Eur Arch Otorhinolaryngol.

[19] Thompson LD, Herrera HB, Lau SK (2017). Thyroglossal duct cyst carcinomas in pediatric patients: report of two cases with a comprehensive literature review. Head Neck Pathol.

[20] Thompson LD, Herrera HB, Lau SK (2016). A clinicopathologic series of 685 thyroglossal duct remnant cysts. Head Neck Pathol.

[21] Li W, Ren YP, Shi YY, Zhang L, Bu RF (2019). Presentation, management, and outcome of lingual thyroglossal duct cyst in pediatric and adult populations. J Craniofac Surg.

[22] Corvino A, Pignata S, Campanino MR (2020). Thyroglossal duct cysts and site-specific differential diagnoses: imaging findings with emphasis on ultrasound assessment. J Ultrasound.

[23] O'Neil LM, Gunaratne DA, Cheng AT, Riffat F (2016). Wide anterior neck dissection for management of recurrent thyroglossal duct cysts in adults. J Laryngol Otol.

[24] Rohof D, Honings J, Theunisse HJ (2015). Recurrences after thyroglossal duct cyst surgery: results in 207 consecutive cases and review of the literature. Head Neck.

[25] Taha A, Enodien B, Frey DM, Taha-Mehlitz S (2022). Thyroglossal duct cyst: a case report and literature review. Diseases.

